# How microrobots should be translated: A clinical and value‐centered readiness framework

**DOI:** 10.1002/btm2.70112

**Published:** 2026-02-03

**Authors:** Hakan Ceylan, Edoardo Sinibaldi, Sanjay Misra, Pankaj J. Pasricha, Dietmar W. Hutmacher

**Affiliations:** ^1^ Medical Microrobots Lab, Department of Physiology and Biomedical Engineering Mayo Clinic Scottsdale Arizona USA; ^2^ Max Planck Queensland Centre Queensland University of Technology Brisbane Queensland Australia; ^3^ Italian Institute of Technology Genoa Italy; ^4^ Department of Radiology Mayo Clinic Rochester Minnesota USA; ^5^ Department of Medicine Mayo Clinic Scottsdale Arizona USA; ^6^ Faculty of Engineering, School of Mechanical, Medical and Process Engineering Queensland University of Technology Brisbane Queensland Australia; ^7^ Australian Research Council (ARC) Training Centre for Multiscale 3D Imaging, Modelling, and Manufacturing (M3D Innovation) Queensland University of Technology Brisbane Queensland Australia; ^8^ Australian Research Council Training Centre for Cell and Tissue Engineering Technologies Queensland University of Technology Brisbane Queensland Australia; ^9^ Centre for Behavioral Economics, Society & Technology (BEST) Queensland University of Technology (QUT) Kelvin Grove Queensland Australia

**Keywords:** medical robot, microrobot, millirobot, technology readiness, translational innovation

## Abstract

Untethered mobile milli/microrobots hold transformative potential for interventional medicine by enabling more precise and entirely non‐invasive diagnosis and therapy. Realizing this promise requires bridging the gap between groundbreaking laboratory demonstrations and successful clinical integration. Despite remarkable technical progress over the past two decades, most millirobots and microrobots remain confined to laboratory proof‐of‐concept demonstrations, with limited real‐world feasibility. Here, we identify key factors that slow translation from bench to bedside, focusing on the disconnect between technical innovation and meaningful patient outcomes. We argue that the long‐term impact and sustainability of the field depend on aligning development with unmet clinical needs, demonstrating feasibility, value and integration potential into existing clinical workflows. To foster translational research of milli/microrobots, we introduce a strategic milli/microrobot Technology Readiness Level framework (mTRL), which maps system development from initial conceptualization to clinical adoption through clearly defined milestones and their associated stepwise activities. The mTRL model provides a structured gauge of technological maturity, a common language for multi‐disciplinary collaboration and actionable guidance to accelerate translational development toward new, safer and more efficient interventions.

## 1 INTRODUCTION

Robotic surgery was once a futuristic idea whose time may never come; today it shapes minimally invasive care. Over the past decade, it has enabled greater precision, reduced tissue trauma, and faster recovery.^1^ Systems such as Intuitive Surgical's Da Vinci have achieved widespread adoption, with over 14 million procedures and more than 76,000 trained surgeons worldwide as of 2025.^2^ Building on this momentum, newer systems have broadened the field: Mako Spine (Stryker) from orthopedics to, more recently, spine surgery^3^; Monarch Platform (Johnson & Johnson) and Galaxy System (Noah Medical) for bronchoscopy^4^, ^5^; and Hugo RAS (Medtronic) emerging as a multi‐specialty platform for urology, gynecology, and general surgery.^6^ Their commercial traction and clinical success continue to drive new investment and research, expanding the ambition of interventional robotics and setting the stage for systems with even greater precision and automation.^7^

Despite the progress, significant anatomical challenges remain in reaching small, tortuous, or highly delicate anatomical regions using tethered robotic instruments. Many vascular, biliary, reproductive, fetal, and intracranial targets are difficult or unsafe to access because forces must be transmitted through long, flexible instrument paths, producing friction, hysteresis, and non‐linear mechanical coupling that degrade precision.^8^, ^9^ In such confined or delicate environments, even small contact forces or shear stresses can provoke endothelial injury, inflammation, or irreversible tissue damage.^10^

These persistent limitations have motivated growing interest in (Box 1) *untethered milli/microrobots*. By operating directly at the target site rather than relying on long mechanical linkages, these miniature mobile devices could navigate narrow, tortuous, or otherwise inaccessible anatomy with substantially less iatrogenic trauma, offering a fundamentally different strategy for reaching small or delicate regions. Over the past three decades, progress in actuation, locomotion, materials, and control have led to the creation of a (Box 2) *toolbox* of small‐scale robotics, with a great number of rigid and soft/deformable body designs, locomotion modes from swimming to rolling, and functions including biosensing, cargo delivery, tissue biopsy, and collective swarm behaviors.^11^, ^12^, ^13^, ^14^, ^15^, ^16^, ^17^, ^18^ These foundational efforts have sparked the emergence of startup companies seeking to pioneer entirely new procedures using milli/microrobots.^19^, ^20^, ^21^, ^22^

BOX 1 **Untethered milli/microrobots**

In this review, a milli/microrobot refers to a complete, clinically deployable platform composed of two inseparable components: (1) the miniature robot body, which acts as the local end effector, and (2) the essential supporting subsystems required for safe and effective operation in patients.

These subsystems include the remote actuation source (e.g., magnetic, acoustic, or optical fields), real‐time imaging and tracking (e.g., ultrasound, fluoroscopy), deployment and retrieval mechanisms, and the software interface that integrates control, visualization, and workflow. Translational readiness therefore depends not on the robot body alone, but on the maturity and interoperability of the entire “turnkey” system within real clinical environments.

For clarity, millirobots refer to mobile robotic devices that typically operate at the sub‐millimeter to millimeter scale (10^−2^–10^−4^ m), where they can be individually tracked with standard imaging and manipulated as discrete tools. Microrobots, by contrast, function at the micrometer to sub‐millimeter scale (10^−4^–10^−6^ m), often in swarms or collectives, and are designed for tasks at cellular or microvascular dimensions. While these classes differ in scale and operating paradigms, both require full‐system integration, validated safety, and workflow compatibility for translational progress. Most prior surveys classify milli/microrobots primarily by locomotion design, actuation mechanism, or materials; here, we emphasize the system‐level definition because translation depends on the maturity of the complete platform rather than on the robot body alone.^11^, ^28^, ^29^

BOX 2 **Toolbox**

In this Review, *toolbox* refers to the growing set of milli/microrobotic capabilities developed through discovery research. These stem from inventive activities in materials, actuation strategies, locomotion modes, control methods, sensing and imaging integration, and fabrication techniques. Collectively, the toolbox enables the design of systems that can be tailored, combined, and matured to meet the demands of specific clinical applications.

Despite this exciting creativity, the transition from laboratory studies to clinical implementation remains slow, fragmented, and immature. Most demonstrations remain proof‐of‐concept studies under tightly controlled experimental conditions in academic laboratory environments, with limited feasibility for deployment in real‐world healthcare settings. Here, we identify the root causes of this innovation‐to‐implementation gap and argue that effective translation requires (Box 3) *value‐centered design* from the outset. This suggests grounding milli/microrobot development on (Box 4) *unmet clinical needs*, practical feasibility, and seamless integration into clinical workflows. To structure this process, we introduce the milli/microrobot Technology Readiness Level (mTRL) framework, which maps system development from concept to clinical adoption through clearly defined milestones and stepwise activities. This roadmap provides actionable guidance for aligning technical innovation with clinical utility, enabling the more effective translation of untethered milli/microrobot technologies from proof of concept to patient impact.

BOX 3 **Value‐centered design**

In this Review, value refers to the net clinical benefit a milli/microrobot provides relative to current standards of care. Value‐centered design begins at the earliest conceptual stages by aligning the robot's intended function, scale, materials, and imaging compatibility with a clearly defined unmet clinical need and the practical constraints of procedural workflow.

As the milli/microrobot system matures with established technical feasibility, value should be progressively demonstrated through measurable evidence of.

- *potential clinical impact* (e.g., reduced tissue trauma, improved targeting, functional benefit),
- *workflow feasibility* (e.g., imaging compatibility, procedure time, operator usability),
- *safety and biocompatibility*, and
- *regulatory and manufacturing readiness* (e.g., sterilization tolerance, reproducibility, GMP feasibility).

Within the mTRL framework, value must be confirmed by the end of Milestone B (mTRL5) through reproducible large‐animal studies showing that benefits meaningfully exceed risks and justify progression toward first‐in‐human evaluation. In this way, value‐centered design operationalizes translation across all stages, not only as a final checkpoint, but as a guiding principle that shapes early design decisions, de‐risks downstream feasibility, and aligns technical innovation with realistic clinical utility.

BOX 4 **Unmet clinical need**

An *unmet clinical need* is a clearly defined patient problem in which current standard‐of‐care options fail to deliver acceptable safety, effectiveness, or access for the intended indication. The need should be supported by clinical evidence and guideline‐based reasoning and framed as a solution‐agnostic capability gap. A precise need statement anchors the translational development of milli/microrobots in real clinical practice, not in abstract technical possibility.

**Scenario 1** | Vascular access.

**Patient**: 68‐year‐old man with critical limb threatening ischemia; angiography shows heavily calcified, tortuous distal tibial/pedal arteries (≈1.5–2.5 mm). Not a surgical bypass candidate.

**Problem**: Multiple failed antegrade crossing attempts; standard 0.014–0.018″ wires/microcatheters skate into the calcified plaque in angulated segments, causing intimal injury/perforation risk.

**Unmet need**: A minimally invasive navigation method that maintains true‐lumen traversal (or controlled subintimal track with predictable re‐entry) through calcified, tortuous distal vessels to develop a definitive endovascular revascularization strategy.

**Robot design implication**: A device with atraumatic, precise navigation capability in tortuous anatomy; built with hemocompatible, low thrombogenic materials; fluoroscopic visibility; robust retrieval strategies.

**Scenario 2** | Fetal intervention.

**Patient**: 24‐week monochorionic–diamniotic twin pregnancy twin‐to‐twin transfusion syndrome (Quintero stage II–III) requiring selective ablation of placental vascular anastomoses.

**Problem**: Current fetoscopes and 8–10 F valved introducers are relatively large/rigid for the chorionic‐plate workspace; transabdominal entry through uterus and membranes increases risk of iatrogenic membrane rupture, chorioamniotic separation, and preterm labor.

**Unmet need**: A low‐profile, low‐trauma method that is image‐guided (ultrasound‐first in current practice) to navigate the amniotic cavity, stabilize on the chorionic plate, and deliver vessel‐selective ablation with tightly limited thermal spread, minimizing membrane injury and large/rigid hardware.

**Robot design implication**: Soft and highly maneuverable robot body with size <2 mm; atraumatic navigation with low contact force/pressure; deployment and retrieval compatible with valved introducer sheaths; image‐guidance ready (ultrasound‐first); selective vessel ablation capability with limited thermal spread (<1 mm).

**Scenario 3** | Gastrointestinal navigation.

**Patient**: 52‐year‐old woman, prior Roux‐en‐Y gastric bypass. CT/MRI shows a 1.5–2.0 cm submucosal lesion in the excluded stomach concerning for early gastric cancer.

**Problem**: Definitive diagnosis requires submucosal tissue. Standard transoral endoscopy cannot reach the remnant; endoscopic ultrasound‐assisted access or percutaneous gastrostomy are possible but carry risks (leak, tract creation in a potentially malignant field, staged procedures). Laparoscopic/transgastric surgery entails re‐entry in a scarred field with higher morbidity.

**Unmet need**: A minimally invasive, low‐trauma intraluminal method that can navigate altered anatomy to reach the excluded stomach and obtain a depth‐controlled submucosal biopsy with hemostasis, while avoiding formal surgery.

**Robot design implication**: A remotely steerable device with size <10 mm; atraumatic navigation with traction on wet mucosa and tight turning radius; adjustable biopsy depth to capture >5 mm mucosal‐submucosal specimen; leak‐proof specimen containment; safe retrieval strategy.

Here, while we illustrate the mTRL framework using mainly magnetically actuated milli/microrobots, currently the most advanced modality with large‐animal demonstrations and clinically compatible actuation systems, the framework itself is modality‐independent. Ultrasound‐driven, optically actuated, chemically propelled, and biohybrid microrobots all hold strong scientific promise, but most remain at early‐stage technical maturity due to foundational limitations in deep‐tissue energy delivery, biochemical safety, immune compatibility, or clinical workflow integration. As such, these platforms cannot yet be meaningfully assessed for translational applications.

**FIGURE 1:**
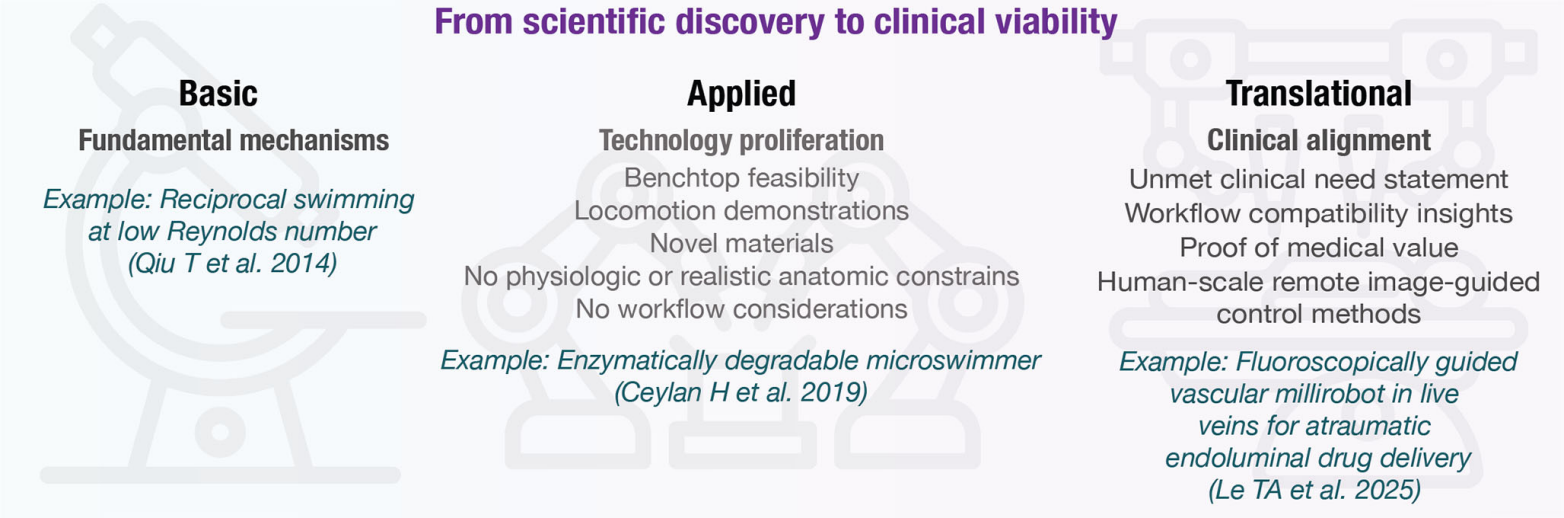
Progress from scientific discovery to clinical viability in small‐scale robotics. Most current milli/microrobot research resides in the Basic and Applied domains, where fundamental mechanisms and benchtop feasibility are explored without direct alignment to clinical constraints. Entry into the Translational domain requires grounding development in a defined unmet medical need and demonstrating workflow compatibility, safety, and potential medical value. By highlighting this distinction, the figure underscores why early integration of clinical considerations is essential for guiding promising concepts toward real‐world impact. (References for Qiu T et al. 2014,^12^ Ceylan H et al. 2019,^23^ Le TA et al. 2025^24^).

Additionally, our intention is not to constrain curiosity‐driven milli/microrobot research, but to clarify how basic and applied discoveries eventually form the foundation for clinically meaningful systems. Most current research resides in the Basic and Applied domains, where the goal is to uncover physical mechanisms, develop new materials or actuation methods, and demonstrate isolated proof‐of‐concept behaviors (Figure 1). These efforts are essential for expanding the design space, but they are generally not anchored to a specific unmet medical need or to the anatomical, imaging, workflow, and safety constraints that govern real procedures.

When the aim is to reach patients, however, development must gradually transition into the Translational domain, where early design choices are shaped by clinical feasibility, regulatory expectations, scalability, and the practical realities of adoption. The timing of this Perspective reflects the fact that several enabling technologies, human‐scale magnetic actuation, improved fluoroscopic and ultrasound visibility, soft materials, and advanced microfabrication have matured to a point where translational considerations are no longer hypothetical but necessary.^24^, ^25^, ^26^, ^27^ A translational framework is therefore introduced not to limit exploratory work but to provide a structured pathway for channeling these emerging capabilities toward clinically viable systems (Boxes 3 and 4).

## 2 WHY TRANSLATION STALLS: THE INNOVATION IMPLEMENTATION GAP

The field of small‐scale robotics remains in the “fluid” phase of the innovation life cycle, characterized by rapid design proliferation and conceptual breakthroughs.^30^ This stage fosters creativity but often comes at the expense of reliability, reproducibility, and scalability. Academic reward structures reinforce this tendency: high publication volume and novelty are more strongly incentivized than durability, system‐level integration, or clinical feasibility.

This culture amplifies claims of clinical potential without comparing to existing benchmarks. Unmet clinical needs are often loosely defined, and safety and efficacy relative to current standards remain untested. Essential system‐level requirements, such as scalable manufacturing, biocompatibility, sterility, reproducibility, and workflow compatibility, are rarely demonstrated. Fragmentation into specialized sub‐domains (materials, actuation, imaging, fabrication) has deepened technical expertise but sidelined integration across the full translational pathway.

The result is a persistent gap between laboratory concepts and clinically implementable solutions, which often leads to inflated expectations and/or misaligned developments. Many milli/microrobots are technically impressive yet “off‐target” for medical application: over‐engineered for feasibility studies but under‐prepared for the practical realities of patient care.^30^, ^31^ Bridging this gap requires rethinking how progress is measured, not just by novelty, but by consistent advancement toward clinically defined value.

## 3 VALUE‐CENTERED DESIGN

Because the development of medical devices and their indications for use are tightly regulated under national oversight, translational small‐scale robotics must be conceived and evaluated with regulation in mind. Effective translation of milli/microrobots begins with a clearly defined unmet clinical need and a framework that prioritizes demonstrable patient value. Technical excellence alone does not guarantee adoption; success requires a systems‐level program that anticipates anatomic, physiologic, and procedural constraints, maps regulatory pathways, and aligns with reimbursement and broader healthcare economics. Early, iterative co‐development with clinicians, including physicians, nurses, and technical staff, is instrumental for shaping indications, workflow integration, training requirements, and long‐term outcomes.

Accordingly, every design choice in a milli/microrobot system must serve a clinically relevant function, contributing to the safety and effectiveness of the care. Robot size, actuation method, and imaging compatibility must reflect the target anatomy while ensuring safe deployment, retrieval, and operator‐friendly use. In practice, barriers such as sterilization, reproducibility, and compliance with hospital standards play the biggest role in determining adoption. Shifting established clinical workflows requires years of cumulative evidence. Empirically, the more complex an innovation, the less likely it is to be adopted, scaled, spread, and sustained in healthcare settings.^32^

Progress with the specific milli/microrobot system will also be benchmarked against the standard of care. Regulatory agencies such as the FDA (USA), EMA (EU), and TGA (Australia) typically require evidence of at least non‐inferiority and ideally superiority in safety and effectiveness relative to existing diagnostics or therapies. Demonstrating credible improvements is critical not only for market authorization but also for clinician adoption and eventually payer reimbursement. Achieving this goal down the road demands establishing feasibility with reproducible validation and demonstrating clinical value in preclinical disease models, regulatory foresight, scalable manufacturing, and system‐level completeness.

Without such alignment, even the most inventive milli/microrobots risk remaining confined to the laboratory, stalling in the “valley of death” where promising concepts fail to become viable clinical solutions.^33^ Innovation studies describe a similar pattern in the Gartner hype cycle: enthusiasm peaks before real‐world evidence accrues, often leading to stagnation or collapse. To avoid this fate, small‐scale robotics field must embed translational considerations early, when expectations are highest. A value‐centered framework anchors robotic engineering to unmet needs of patients, day‐to‐day care delivery, and the emerging technology's downstream effects on outcomes, costs, staffing, and logistics over years. The reflection of these effects should also be measured transparently and guided insightfully, thereby motivating a dedicated mTRL as a shared yardstick for translational maturity of translational milli/microrobots.

## 4 TECHNOLOGY READINESS FRAMEWORK FOR MILLI/MICROROBOTS

Technology Readiness Level (TRL) scales are widely used across aerospace, automotive, and energy sectors to assess technology maturity.^34^, ^35^, ^36^ Over time, TRL has evolved into an objective, evidence‐based tool for guiding product development and integrating innovations into complex systems.^32^ Recognizing the differing demands of each sector, field‐specific TRL scales have emerged.^33^, ^34^, ^35^ Aligning development plans to TRLs reduces financial and coordination costs, improves time efficiency, and manages risk, which are critical advantages in the regulated medical device market.^37^ For example, the U.S. Department of Defense employs comprehensive Technology Readiness Assessment guidelines to control cost growth in new technology initiatives.^38^

Small‐scale robotics would benefit significantly from a TRL scale formulated explicitly for translational development. In addition to the general challenges common to medical device development, untethered milli/microrobots face a set of translational dependencies that are fundamentally different from those addressed by existing TRL variants. These include: (i) human‐scale remote actuation capabilities compatible with clinical imaging suites; (ii) robust visibility within body, localization, tracking and navigational strategies; (iii) reliable deployment, retrieval and contingency mechanisms tailored to intended use environments; (iv) unique toxicity, hemocompatibility and immunological considerations arising from mobile devices operating in blood, mucus‐lined or dynamically deforming tissues; and (v) autonomous or semi‐autonomous navigation under physiologic dynamics such as pulsatile flow, vasospasm, organ motion and variations in tissue compliance. These domain‐specific constraints represent translational bottlenecks that classical TRL systems, and existing micro/nanorobotics roadmaps, do not systematically capture, underscoring the need for a field‐specific readiness framework such as mTRL.

A shared, domain‐specific rubric would also improve communication among cross‐disciplinary research teams, clinicians, regulators who may be unfamiliar with the emerging technology's unique features, and investors focused on milli/microrobot systems. For example, an engineer developing a soft endovascular millirobot may judge the system ready for animal testing based on benchtop navigation, while an interventionalist would require demonstrated fluoroscopic visibility, predictable retrieval, and compatibility with standard guidewire access. The mTRL framework codifies these cross‐disciplinary expectations into concrete milestones, preventing premature progression.

Our mTRL framework combines two complementary dimensions: *technical maturity—evidence* that capabilities, such as remote robot actuation, control, imaging compatibility, perform in environments of increasing realism—and *clinical alignment—evidence* that those capabilities can create a clinical value by satisfying a defined medical scenario, including anatomical access, disease indication, procedural constraints, and workflow integration. This dual‐axis assessment extends beyond isolated laboratory performance to capture real‐world readiness, thereby reducing development risk and directing investment toward applications with the greatest translational potential. It also helps calibrate expectations within the field by highlighting the clinical drivers behind research, the incremental steps required, and discouraging premature or inaccurate claims, which we believe is also essential for public relations.

**FIGURE 2:**
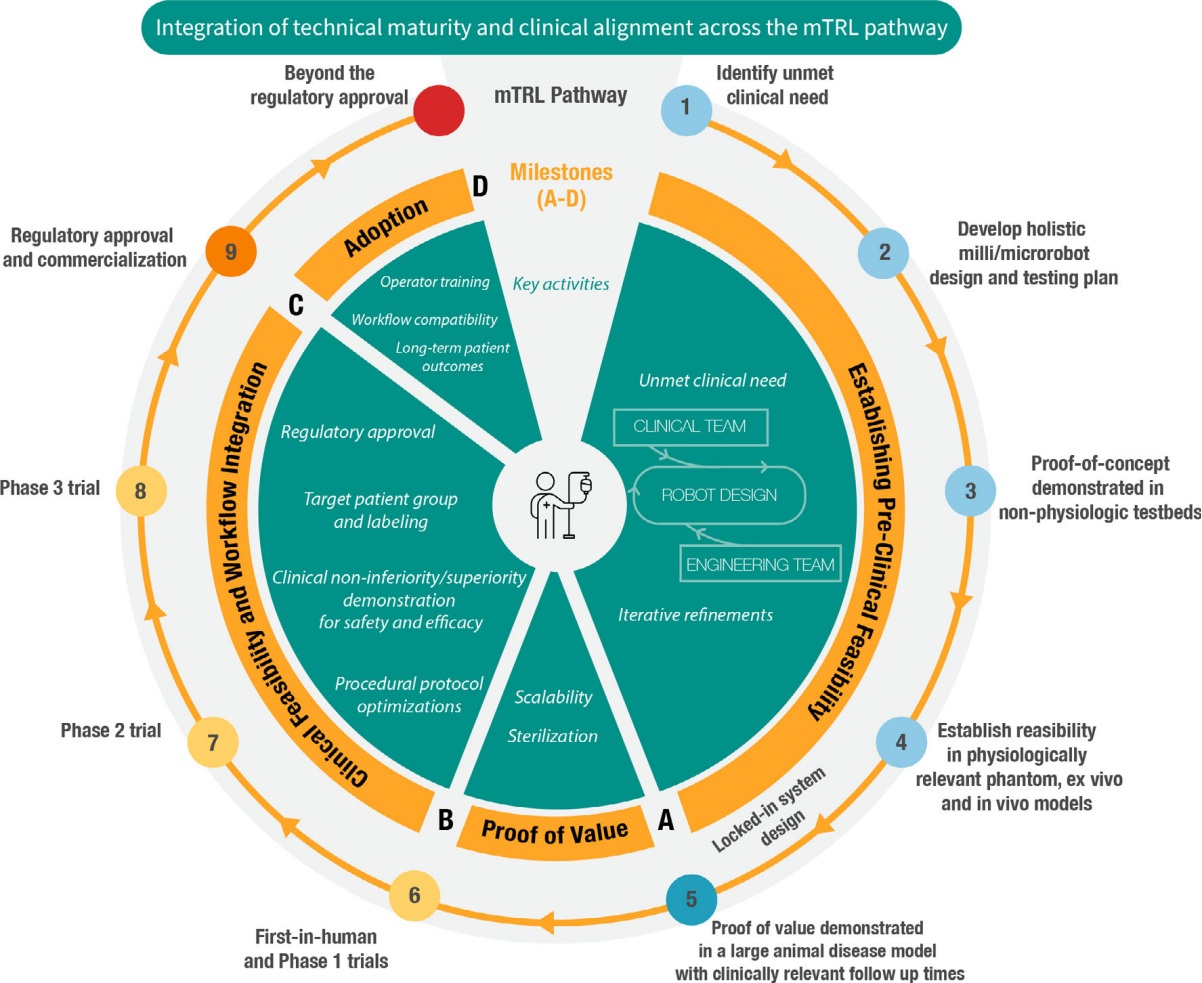
Value‐based translation roadmap of untethered milli/microrobots. Translating milli/microrobots from benchtop proof‐of‐concepts into clinically adopted technologies requires aligning technical innovation with unmet clinical needs, feasibility constraints, and workflow integration. The milli/microrobot Technology Readiness Level (mTRL) pathway organizes this progression from identifying a specific unmet clinical need (mTRL1) through preclinical feasibility (mTRL2‐4), proof of medical value in large‐animal disease models (mTRL5), first‐in‐human evaluation (mTRL6), Phase 2–3 trials (mTRL7‐8), and commercialization (mTRL9) and eventual clinical adoption. Clinical alignment begins at mTRL1 and constrains all subsequent design decisions, locking in system architecture early and necessitating iterative refinement across mTRL1‐4 before feasibility and proof‐of‐value can be demonstrated. Progress across the mTRL pathway depends on continuous interaction among key stakeholders: clinicians (defining the unmet need, procedural constraints, and workflow requirements), engineers (developing systems compatible with anatomic, physiologic, and imaging realities), regulatory bodies (ensuring safety and compliance), patients (shaping outcome priorities and perception), and market stakeholders (enabling sustainability and reimbursement). Together, these contributors create a dynamic feedback loop in which insights at each stage refine earlier decisions, increasing the likelihood that emerging milli/microrobot systems become clinically relevant, adoptable, safe, and sustainable.

The mTRL organizes development into four clinical milestone phases comprising nine readiness levels (Figure 2). Table 1 summarizes each level's objectives, typical approaches, and Go/No‐Go criteria indicating the evidence thresholds that must be met to progress to the next mTRL stage; failure to meet them requires redesign or further testing. We structure mTRL with the expectation that most milli/microrobot systems will follow a medical device regulatory pathway. Systems whose primary mode of action is metabolic or biochemical may fall under pharmaceutical regulation.^39^ Some may be classified as combination products that integrate a delivery robotic device with therapeutic cells or drugs.

**TABLE 1:** mTRL framework for translating milli/microrobots.

mTRL	Description	Objective	Key activities	Go/No‐Go Criteria
1	Unmet clinical need defined	Identify a clinically meaningful unmet need	Review clinical workflows and current standard of care Engage clinicians to characterize challenges and current failure points Map limitations of existing tools and identify failure points	Unmet need is clearly articulated Evidence‐based rationale for pushing a medical/microrobot solution
2	Holistic milli/microrobot system plan developed	Formulate a clinically aligned milli/microrobot system concept	Assemble a multi‐disciplinary team (clinicians, engineers, imaging experts, regulatory advisors) Define candidate designs, materials, actuation, control and imaging strategies Incorporate anatomic, physiologic and procedural constraints Identify early safety and failure‐case risks	Coherent system architecture (robot body, materials, fabrication, imaging, tracking, actuation) Development plan aligned with regulatory and manufacturing feasibility
3	Proof‐of‐concept in non‐physiologic (in vitro) testbeds	Demonstrate basic function under controlled, non‐physiologic conditions	Benchtop and simplified phantom testing of milli/microrobots Iterative prototyping and subsystem integration Preliminary imaging visibility and actuation robustness assessment Early biocompatibility and material safety screening	Reproducible system‐level behavior under controlled conditions Defined performance endpoints (navigation, visibility, actuation, safety screens) for later preclinical studies
4	Feasibility in physiologically relevant models	Validate performance under realistic anatomic and physiologic conditions	Test in physiologically relevant phantoms, ex vivo tissues, and small animals Validate imaging visibility, navigation and actuation under realistic conditions Define feasibility, reproducibility and scaling thresholds Begin workflow integration with imaging/actuation platforms Refine through mTRL 1–4 as needed	Demonstrated navigation, visibility, and function under realistic constraints Draft design history and regulatory documentation initiated (design file, preclinical protocols outline)
5	Proof of clinical value in large‐animal disease models	Demonstrate safety and functional benefit relative to current practice	Large‐animal studies with clinically relevant follow‐up Scale actuation/imaging hardware to clinically realistic ranges Refine procedural workflow and operator interactions Define Go/No‐Go criteria	Clinically relevant improvement in functional endpoints in disease models Locked system design and regulatory pre‐submission preparation completed
6	First‐in‐human and Phase 1 trials	Establish initial human safety, usability, and workflow compatibility	Secure regulatory approval for first‐in‐human use Conduct first‐in‐human and Phase 1 evaluations Assess usability, workflow compatibility, and imaging/actuation performance Identify required refinements before advancing to Phase 2	Acceptable safety profile in humans Performance consistent with preclinical data Data package adequate to design a Phase 2 trial
7	Phase 2 clinical trials	Evaluate safety and preliminary efficacy in target population	Controlled study in moderate‐sized patient cohort Define and evaluate clinical endpoints in consultation with regulators Refine milli/microrobot control parameters, imaging strategy and operator performance	Evidence supporting progression to pivotal Phase 3 trial Validated procedural workflow and refined robot use parameters
8	Phase 3 clinical trials	Confirm risk–benefit and comparative performance relative to standard of care	Conduct statistically powered, multicenter trials across diverse sites Confirm reproducibility of performance and workflow integration across operators Finalize labelling indications for use, and risk‐mitigation protocols	Demonstrated clinical superiority, non‐inferiority, or added value sufficient for regulatory approval Reproducible safety and efficacy across centers
9	Regulatory approval and commercialization	Achieve clinical adoption and demonstrate long‐term safety, usability and effectiveness in real‐world practice	Manufacturing and distribution of milli/microrobots under full GMP compliance Post‐market surveillance, vigilance reporting, and periodic safety updates Refinement of procedural workflows and integration into hospital systems Standardized training, credentialing and support programs for clinicians Minor robot refinements and software updates within the approved design envelope	Regulatory approval and maintained compliance with post‐market requirements Established reimbursement pathways and sustainable commercial model Demonstrated real‐world safety, effectiveness and clinician adoption

### 4.1 Milestone A (mTRL1‐4): Identifying unmet clinical need and establishing preclinical feasibility

Milestone A represents the first translational checkpoint. It is reached once a proposed milli/microrobot system has progressed from concept generation (mTRL1) to feasibility demonstration in physiologically relevant models (mTRL4). Achieving this milestone requires iterative refinement of a design concept that carefully addresses unmet clinical needs and is capable of operating under increasingly realistic conditions.

Translational research starts with the identification of a specific clinical problem and an innovative milli/microrobot concept developed to address it. At mTRL 1, this is achieved through literature review, initial market surveys, and in‐depth discussions with clinicians, surgeons, and other healthcare professionals to understand both the medical need and the landscape of current solutions. The specific aim is to articulate a medically grounded rationale that justifies a milli/microrobot concept that can be potentially superior to the existing standard of care.

At mTRL2, the vision develops into a system‐level plan. Alternative designs, materials, actuation strategies, and control methods are defined, while physiologic and anatomic constraints are incorporated from the outset. Early design choices must already reflect regulatory foresight and fabrication scalability to support eventual Good Manufacturing Practice (GMP) feasibility, since downstream changes can be both costly and prohibitive for translation. Although prototyping is still performed in phantoms, ex vivo tissue, or small animals, development should increasingly converge toward conditions that simulate an operating room environment.

At mTRL3, controlled non‐physiological in vitro experiments (e.g., Petri dish or simplified flow channels) verify that the robot functions reproducibly as a complete system. Iterative prototyping and optimization ensure that all components work seamlessly together, while extensive material and mechanical characterization provides the baseline for safety and performance. Endpoints such as navigation accuracy, imaging visibility, actuation robustness, and nontoxicity are defined here, laying the foundation for subsequent preclinical evaluation.

At mTRL4, testing progresses to physiologically relevant phantoms, ex vivo tissues, and small‐animal models. This stage is where robust iteration truly takes hold: performance is validated under increasingly realistic anatomic and pathophysiologic conditions, reproducibility is established, and safety endpoints are refined. Results from this stage form the specific design files and standard operating protocols needed to justify large‐animal studies and provide the evidence base for moving to higher technology readiness level.

### 4.2 Milestone B (mTRL5): Demonstrating the proof of clinical value

While Milestone A focuses mainly on establishing feasibility through iterative refinement of the milli/microrobot system, Milestone B shifts the emphasis to demonstrating clinical value. At this stage, the locked‐in lead system design and operational protocols from mTRL4 are evaluated across the full interventional process, asking whether the approach would justify its risks and benefits if applied to patients.

For most researchers in small‐scale robotics, the familiar territory ends at mTRL1‐4: benchtop proof‐of‐concepts, phantom models, and small‐animal studies. The transition to mTRL5 represents the most significant leap, requiring demonstration of safety, efficacy, and functional value in large‐animal disease models that closely replicate human anatomy, pathophysiology, and procedural workflows. These studies provide insight into whether the intervention can alter disease progression, withstand real‐world procedural challenges, and generate clinically relevant outcomes within follow‐up times comparable to human care.

Achieving this level requires a fully integrated, scalable system and close collaboration with medical teams. Hospital integration plans must also come into sharper focus, covering imaging compatibility, workflow logistics, staff training, and patient safety protocols.^40^ Importantly, the risk–benefit balance must be made explicit, weighing improvements in safety and efficacy against the economic realities of healthcare delivery.

In practice, achieving Milestone B introduces substantial logistical, ethical, and financial challenges: large‐animal housing, surgical support, medical imaging access, and regulatory compliance demand resources. These resources are not typically available at the direct disposal of a single academic lab working on robots. Anticipating these requirements underscores the need for partnerships with clinicians and clinician scientists, regulatory experts, and industry stakeholders needed to move forward with appropriate resources.

### 4.3 Milestone C (mTRL6‐8): Frist‐in‐human, clinical trials, and regulatory approval

Once mTRL5 is achieved, the regulatory pathway needs to be clarified in consultation with the relevant authorities, considering the intended use, risk profile, and device classification. In the United States, the 510(k) process allows a device to enter the market by demonstrating substantial equivalence to a legally marketed predicate, often without the need for clinical trials. However, first‐generation untethered milli/microrobots are a highly novel device, which will likely require extensive clarification, so the 510(k) pathway would rarely apply. We anticipate that most first‐generation milli/microrobots would follow the Class III medical device route, requiring mTRL6 first‐in‐human trials. These small, tightly controlled studies focus on safety and initial performance, often revealing necessary refinements before broader testing. So far, no human data was shown with milli/microrobots.

As the design variety is diverse in the field of small‐scale robotics, in some cases, milli/microrobots may not fit existing classifications. The FDA's De Novo pathway offers an alternative for novel devices that lack a suitable predicate but do not pose the highest risk. For example, in 2020, AnX Robotica's NaviCam™ Magnetically Controlled Capsule Endoscopy System was granted De Novo classification. As autonomy, actuation methods, and therapeutic capabilities evolve, entirely new device categories may be required to address the unique risks and benefits of these systems.

At mTRL7, Phase 2 clinical trials expand to moderate‐sized patient cohorts, generating statistically meaningful safety and efficacy data while optimizing procedural protocols and training programs. This stage may also compare the device's performance directly to standard‐of‐care alternatives.

mTRL8 involves large‐scale, multicenter Phase 3 clinical trials to confirm the device's overall risk–benefit profile, establish clinical superiority (or non‐inferiority) over conventional treatments, and provide definitive data for regulatory approval and market entry.

### 4.4 Milestone D (mTRL9): Clinical adoption and continuous improvement

mTRL9 is reached when the system is commercially available and integrated into routine clinical workflows, supported by reimbursement mechanisms and post‐market surveillance. Market approval is not the end of development, nor will it guarantee clinical impact. Minor updates (e.g., software refinements) may occur within mTRL9, while major changes in indication, materials, or fundamental design would require revisiting earlier mTRL stages. Long‐term success depends on physician acceptance, ease of training, safety perception, and cost justification. Continuous monitoring of real‐world performance ensures that the device evolves in line with clinical needs while maintaining compliance with regulatory and manufacturing standards.

## 5 ENABLERS OF MILLI/MICROROBOT SYSTEMS IN THE mTRL FRAMEWORK

This section identifies the cross‐cutting enablers that must mature in concert for milli/microrobot translation and maps their maturation in the mTRL roadmap. The core domains are: (i) mechanical robot design; (ii) robotic materials, fabrication, and biocompatibility strategies; (iii) human‐scale, image‐guided actuation and interventional automation; (iv) imaging, localization, and tracking; (v) deployment, retrieval, and contingency planning; and (vi) standardized methods under a quality management backbone. Across mTRL1‐4, the central challenge is progressively integrating these key domains into a coherent, testable system. Because early choices cascade through all later stages, alignment with clinical workflow, regulatory expectations, and usability must be built in from the outset.

### 5.1 Mechanical robot design: Architectural non‐modularity and early design lock‐in

A fundamental distinction between milli/microrobots and conventional medical devices is their near‐complete lack of modularity. Traditional devices, such as guidewires, catheters, endoscopes, and large surgical robots, are composed of separable subsystems whose materials, coatings, and components can be iteratively adjusted late in development without altering the underlying device architecture or regulatory classification.

**FIGURE 3:**
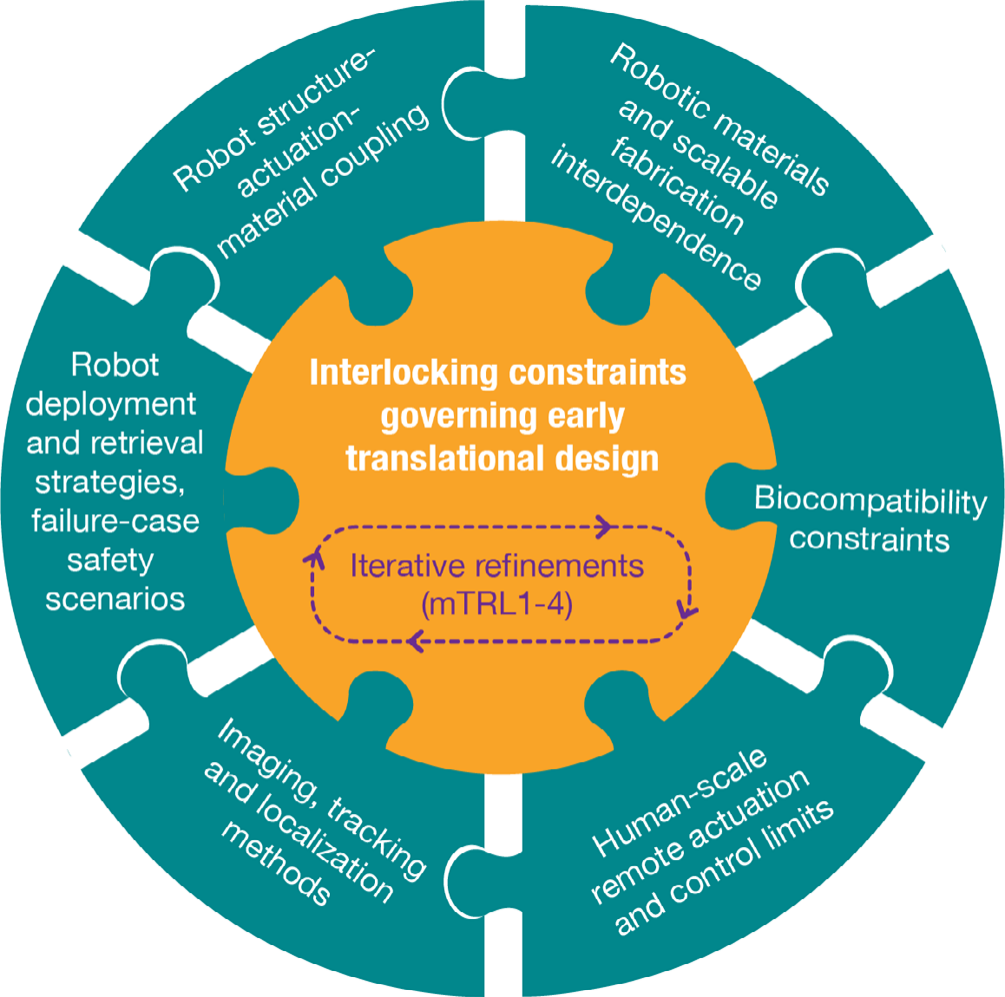
Architectural non‐modularity in milli/microrobot design necessitates early, clinically aligned system integration. Designing a medical milli/microrobot is analogous to assembling a tightly interlocking puzzle in which geometry, materials, magnetic loading, mechanical compliance, imaging visibility, biocompatibility, actuation method, deployment strategy, retrieval options, and failure‐case behavior are all mutually dependent. Because these elements collectively determine robot performance, safety, and manufacturability, altering any one parameter can propagate system‐wide effects. Even seemingly small changes, such as a shift in imaging modality, magnetic content, or fabrication method, can cascade across mTRL1–4 and require redesign and revalidation. Once a system architecture is assembled around a specific clinical need, flexibility for major modifications becomes limited. This inherent non‐modularity underscores why early clinical alignment, regulatory foresight, and workflow compatibility must shape the initial design blueprint for translational milli/microrobot.

By contrast, milli/microrobots derive their functional behavior from tightly coupled physical interactions between geometry, materials, magnetic loading, mechanical compliance, and surface chemistry. This *physical intelligence* means that actuation, locomotion, imaging visibility, biocompatibility, and degradation kinetics are inherently interdependent.^41^ As a result, even small modifications often propagate changes across the entire system, frequently necessitating a full redesign rather than a modular substitution. Figure 3 illustrates this interlocking *design puzzle*: the non‐modularity makes early‐stage design alignment (mTRL1‐4) far more consequential than in traditional medical device development and underscores the need for a dedicated translational readiness framework tailored to the small‐scale robotics. This high degree of interdependency is why clinical alignment must occur at the earliest design stages and why translational progress cannot be retrofitted onto late‐stage benchtop prototypes.

Size is a first‐order decision set by the target anatomic site and, in turn, bounds the engineering toolbox–compatible imaging, actuation options, and feasible locomotion.^14^ Potential areas (nervous, respiratory, urinary, cardiovascular) offer accessible pathways from a few micrometers to a few millimeters. Distal arteries and veins often present ~1–4 mm lumens suited to millirobots, whereas pulmonary microvasculature approaches 2 μm for microrobots. The navigable space in the body is not empty. It is usually fluid‐filled, mucus‐lined, and the geometric structure can be highly patient‐specific. In blood vessels, while arterial cross sections are close to circular, veins are typically elliptical. Vessels are not static structures as described in most of the phantom models used in milli/microrobots. They can deform dynamically in response to pulsation, vasodilation, and vasoconstriction. Consequently, endovascular designs must conform to changing lumen geometry and remain atraumatic to the endothelium, since the preservation of this thin tissue is highly critical for vascular health and inflammation.^24^ This way a milli/microrobotic drug delivery system can also demonstrate a critical medical superiority over existing devices, such as drug‐coated balloons for the local management of vascular inflammation—a major unmet need. Propulsion and control (e.g., magnetic torque) must be scaled against competing local tissue forces, such as mechanical tissue compression, viscous drag, and surface friction and verified for feasibility and reliability in more realistic phantom, ex vivo and in vivo testbeds.

### 5.2 Robotic materials, fabrication, and biocompatibility strategies

Material selection is one of the earliest and most consequential decisions, as it determines the microfabrication methods, device functionality, performance limits, and biocompatibility risks. Although a broad arsenal exists, including photolithography, electroplating, soft lithography, micromolding, two‐photon micro‐printing, laser micromachining of foils, thin‐film deposition/etching, and micro‐assembly, each method imposes constraints on material compatibility, achievable feature size, surface finish, multi‐material joining, sterilization tolerance, fabrication throughput, and cost.^26^, ^42^, ^43^ Devices must be tested for biocompatibility in their intended application context and planned exposure duration. For example, neodymium‐iron‐boron (NdFeB) fillers are widely used because they enable strong remanent magnetization, programmable dipoles, and reliable wireless actuation for precise navigation.^24^, ^44^ They can be acceptable for temporary interventions if fully encapsulated and leakage‐proof. Because NdFeB is corrosion‐prone and can release ions, it is generally unsuitable for permanent implantation. Barrier coatings (e.g., parylene C, silicon oxide, Au/Ti) can provide short‐term corrosion resistance, but their integrity must be verified under sterilization, mechanical wear, and relevant bodily fluids. Applications involving blood contact additionally require thorough hemocompatibility testing, as mobile microrobots may trigger hemolysis, platelet activation, or thrombosis in ways distinct from conventional devices. These evaluations should follow ISO standards (e.g., ISO 10993‐1 for general biocompatibility and ISO 10993‐4 for blood interactions).^45^ For robots intended for long‐term exposure, immunological compatibility becomes critical, since chronic inflammation or foreign body responses can compromise both functionality and patient safety.^46^ Robot design features such as surface texture, body structure, porosity, and degradation behavior must therefore be evaluated case‐by‐case to minimize adverse responses for the target tissue. If retrieval is uncertain, engineered biodegradation may be considered, but only with controlled fragment size and non‐toxic products.^23^ Radiopaque and echogenic nanoparticles incorporated into the robot body frame provide visibility with fluoroscopic and ultrasound imaging, respectively, and thereby estimate robot pose for reliable locomotion guidance.^47^ Finally, sterilization compatibility (gamma irradiation, ethylene oxide treatment, low‐temperature plasma) must be confirmed without causing demagnetization, swelling, embrittlement, and so forth.

### 5.3 Human‐scale actuation

A critical bottleneck for clinical translation lies in human‐scale actuation. The overwhelming majority of magnetic milli/microrobots to date have been demonstrated in laboratory settings using benchtop electromagnetic coils or fixed permanent magnets. While these systems provide valuable proof‐of‐concept control, they do not scale easily to large‐animal or human procedures. Recently, a few human‐scale magnetic control approaches were reported using coil arrays^48^ and robotic permanent magnet systems integrated with open‐space imaging modalities.^47^, ^49^, ^50^ These platforms can safely generate magnetic fields in the 5–50 mT range throughout the body, a level sufficient for steering many magnetic milli/microrobots in clinically relevant environments.^24^

For translational research, each approach carries distinct trade‐offs. Coil‐based systems may offer higher flexibility and programmable field control. Still, their complexity, potential cooling challenges in continuous operation, and additional infrastructure costs may create a barrier to widespread adoption in hospital settings. By contrast, permanent magnet systems are more compact and cost‐effective. Mounted on robotic arms, permanent magnets can be programmed to move around the patient without disrupting concurrent imaging, providing a pragmatic pathway toward clinical integration. However, moving mechanical parts and large magnets around the patient and other medical equipment might challenge workflow integration. Systems that align with existing surgical suites and integrate seamlessly with imaging equipment are most likely to lower adoption barriers. Within the mTRL framework, human‐scale actuation represents a decisive checkpoint in transitioning from laboratory‐scale demonstrations to demonstrating potential clinical value.

### 5.4 Control methods: The case for interventional automation

Untethered milli/microrobot systems represent the extreme of surgical miniaturization, where precise actuation, limited onboard sensing, indirect visualization, and nontrivial environmental dynamics (e.g., pulsatile flow, vasospasm, tissue deformation) converge. These characteristics challenge purely manual operation and motivate the use of interventional automation. Milli/microrobots within the human body can demand continuous, high‐precision input under indirect visualization, such as fluoroscopy. This can raise (i) safety risks from device loss or unintended tissue contact; (ii) longer procedure times; and (iii) cumulative radiation exposure for patients during x‐ray‐guided interventions (ALARA principles). For tasks such as those requiring complex soft robot kinematic controls, navigation, or swarm dispersion management, computer control can execute repeatable micromotions faster and more reliably, enforce hard regional safety limits within the tissue, reduce total procedure time, patient exposure to radiation, and the operator's cognitive load. The interventionalist retains live monitoring with instant override. The level of autonomy should be risk‐based, conditioned by anatomy, physiology, imaging latency, and robot design—ranging from fully manual through assistive/shared control to supervised task autonomy and, only when validated, full autonomy.^51^

### 5.5 Imaging, localization, and tracking

Because untethered milli/microrobots typically lack onboard sensing, intraoperative imaging serves as the real‐time feedback loop for localization and control, making the robot's clinical viability inseparable from imaging performance and workflow compatibility. Seamless compatibility with FDA‐approved, widely available imaging modalities already present in hospitals, such as fluoroscopy and ultrasound, is strategic for translational progress. Leveraging existing platforms ensures workflow feasibility and avoids the prohibitive costs of building entirely new infrastructures. Nevertheless, major technological gaps remain: no single modality currently provides the ideal combination of fast acquisition, deep tissue penetration, and high‐resolution anatomical context needed for the safe real‐time tracking and steering of milli/microrobots. Fluoroscopy can visualize robots across a wide size range (0.1–10 mm), while lacking detailed anatomical context and three‐dimensional localization. In addition, prolonged fluoroscopic imaging introduces nontrivial radiation exposure for both patients and clinical staff, which constrains procedure duration and limits the practicality of relying on continuous x‐ray guidance for real‐time robot control. Therefore, safe navigation remains limited. Ultrasound struggles in regions near bone or air cavities, limiting many intracranial and gastrointestinal applications.

Translational research should focus on developing new approaches to alleviate such technical gaps for effective in vivo robot control. A recent emerging concept to this end has proposed integrating fluoroscopic guidance with a virtual reality (VR) environment that houses a digital twin of the operational workspace and a robot avatar.^47^ This visual enhancement approach allows robust tracking and steering with minimal latency, while providing clinicians with an augmented anatomical view. Such frameworks could also enable the integration of additional intraoperative data streams, such as robot velocity, blood flow dynamics, or periodic organ motion, into real‐time control, potentially improving both safety and precision.

Computational approaches integrated into digital twin environments may also alleviate exposure constraints. By using predictive algorithms that fuse intermittent imaging with real‐time estimations of robot pose, blood‐flow dynamics, and physiologic motion, such systems could reduce reliance on continuous fluoroscopy. This would enable safer and smoother control loops, lower radiation burden while maintaining precise navigation.

While promising, these innovations themselves must still be judged against translational criteria: interoperability with existing clinical workflows, reproducibility in physiologically relevant settings, and regulatory acceptance. Within the mTRL framework, such concepts exemplify how emerging technologies can accelerate feasibility if they are developed not as stand‐alone research demonstrations but as integrated, workflow‐compatible solutions.

### 5.6 Deployment and retrieval strategies

Deployment and retrieval remain underexplored but decisive for clinical adoption. For endovascular robots, delivery catheters can facilitate controlled deployment, while retrieval may involve reversing the robot into the catheter or employing magnetic retrieval catheters.^52^ When retrieval is not feasible, biodegradable designs provide an alternative path, ensuring safe elimination from the body and reducing long‐term risks, such as inflammation or fibrosis.^23^ Each strategy entails trade‐offs in terms of safety, feasibility, and regulatory acceptance, which must be aligned with the intended application. For example, while biodegradability can mitigate chronic exposure risks, in vascular settings it may also increase the danger of embolization, requiring careful material and design choices.

### 5.7 Safety considerations, worst‐case scenarios, and failure modes for untethered milli/microrobots

Safety considerations, worst‐case scenarios, and failure modes represent a critical but often underexamined aspect for the current state of milli/microrobots research. Because untethered milli/microrobots lack the physical constraint and operator anchoring of catheter‐based systems, their safety profile is dominated by risks that stem from being freely mobile within the body. These include migration, transient loss of visibility, uncertain retrieval pathways, and sensitivity to physiologic forces, leading to failure modes distinct from those seen in tethered devices.

Because design elements, including geometry, materials, magnetization, surface chemistry, and mechanical compliance, are tightly interdependent, failure modes emerge as system‐level events rather than isolated component faults, and late‐stage fixes usually require rebuilding the system entirely. The most consequential translational failures fall into several measurable categories, including:

*Device loss or uncontrolled migration*: Loss of the robot in complex anatomy, particularly under pulsatile flow, vasospasm, or unexpected branch paths, represents a high‐risk failure mode. Acceptable migration probability and reliable retrieval pathways must be demonstrated before advancing beyond mTRL4.

*Imaging and localization failure*: Transient loss of fluoroscopic or ultrasound visibility, insufficient signal‐to‐noise ratio (SNR), or pose‐tracking drift can lead to uncontrolled motion or prolonged fluoroscopy. These risks motivate quantitative visibility thresholds, e.g., minimum SNR or maximum permissible occlusion time, for safe navigation.^47^

*Actuation failure under physiologic forces*: Magnetic forces that suffice in benchtop studies may fail against tissue deformation, blood flow, peristalsis, or increased depth in large‐animal anatomy. Feasibility requires force‐margin analysis demonstrating that actuation exceeds peak physiologic or supraphysiologic loads with adequate safety margin.^27^

*Manufacturing and sterilization vulnerabilities*: Small deviations in dimensions, magnetization strength, coating thickness, or sterilization‐induced degradation can propagate into large behavioral differences at the microscale. Batch‐to‐batch tolerances and sterilization robustness become quantifiable quality gates at mTRL4–5.

*Workflow and usability failures*: Excessive navigation time, steep operator learning curves, or incompatibility with imaging suites or operating room logistics can render otherwise functional systems impractical. Quantitative benchmarks such as maximum acceptable procedure time or minimum operator performance consistency will eventually be required.

These worst‐case scenarios clarify why milli/microrobot translation requires a domain‐specific readiness framework. Each failure type corresponds to specific Go/No‐Go criteria within the mTRL pathway, particularly across mTRL1–5 where design lock‐in occurs, enabling structured mitigation.

### 5.8 Quality management system for the standardization of methods

Another decisive step moving beyond Milestone B is complying with the increased volume and precision standards of documentation. A Quality Management System (QMS) or equivalent structured framework provides standardized, auditable processes across the product lifecycle, from design and prototyping to manufacturing and testing. Frameworks such as ISO 13485 or the FDA's Quality System Regulation form the basis for regulatory credibility. While full QMS implementation is mandatory for companies, adopting QMS‐aligned practices in academic translational research can prevent costly repetition and streamline the path to clinical trials.

## 6 ECONOMICS OF TRANSLATION

Translational innovation is at least an order of magnitude more expensive than basic or applied research. In the United States, developing a novel Class III therapeutic device from concept to FDA approval typically costs around $54 million, with costs ranging from $25 million to $200 million.^53^ For milli/microrobots, these financial demands would accumulate gradually across multiple technology readiness levels, with distinct cost drivers, funding models, and risk profiles at each mTRL stage.

At early mTRL levels (mTRL1‐5), costs are driven primarily by prototyping, benchtop validation, and small and large animal studies. Funding typically comes from academic grants (e.g., NIH R01 in the U.S., ERC in Europe, NHMRC/ARC in Australia, KAKENHI in Japan, or NSFC in China), philanthropic foundations, or seed accelerators. Although prototypes remain artisanal and need not yet meet regulatory‐grade manufacturing standards, system‐level complexity creates the main financial burden when assembling a milli/microrobot operational platform as described in Figure 3.

A decisive inflection point would occur at and after mTRL5, as systems approach “first‐in‐human” readiness (mTRL6). Costs will increase sharply due to the need for Good Manufacturing Practice (GMP) production, Good Laboratory Practice (GLP) safety testing, and formal engagement with regulators through pre‐submission meetings or Investigational Device Exemption applications. Academic funding for translational research still covers these activities, while strategic industry partnership is also becoming necessary for additional capital investment. Typical sources include early‐stage venture capital or strategic industry partnerships. Establishing reproducible GMP manufacturing, consistent material properties, validated magnetic/mechanical performance, and reliable sterilization protocols often represents one of the largest engineering costs before clinical trials.

Expenses will escalate further during first‐in‐human trials, which require hospital integration, Institutional Review Board approval, and compliance with clinical‐grade quality systems (e.g., ISO 13485). Additional hidden costs will arise from workflow adaptation, staff training, increased procedure times, and potential modifications to imaging infrastructure. Funding at this stage for medical devices often depends on venture capital, government translational initiatives, or public‐private consortia, such as ARPA‐H initiatives.^54^

The largest financial burden is anticipated at later mTRL stages during multicenter Phase 2–3 trials and large‐scale GMP manufacturing required for commercialization. Here, payer acceptance and reimbursement considerations play a decisive role: milli/microrobots will compete economically with established alternatives such as catheters, stents, or tethered robotic systems. Demonstrating clear value, clinical superiority, non‐inferiority at lower cost, or new therapeutic capability is essential for both regulatory approval and adoption.

## 7 KEY LESSONS FROM OTHER TRANSFORMATIVE MEDICAL DEVICE ADOPTION

The history of successful medical devices challenges the often‐attributed Henry Ford quotation, “If I had asked people what they wanted, they would have said faster horses.” In healthcare, value pull rather is a more dominant theme than a technology push, meaning that adoption follows when innovations deliver demonstrable patient benefits, integrate into clinical practice, and build trust. Transformative technologies such as coronary stents, pacemakers, cochlear implants, and Da Vinci robot were not adopted merely because they were disruptive; they succeeded because they solved big unmet clinical needs in reliable and measurable ways, the result of years of focused, systematic, and iterative development around clearly defined problems.

The history of such medical device innovation offers valuable insights into the translational trajectory of milli/microrobots. Landmark cases reveal recurring themes that determine whether what is often described in publications as technically novel devices may ultimately succeed in clinical practice.

### 7.1 Workflow integration is decisive

The widespread adoption of coronary stents was driven not only by their ability to reduce restenosis but also by their seamless incorporation into a catheter‐based infrastructure that had been developed over decades, spanning angiography, fluoroscopic imaging, and balloon angioplasty since the late 1970s.^55^ Because stents could be deployed using the same interventional tools and techniques already familiar to physicians, they required no radical reorganization of practice. This continuity greatly accelerated their acceptance in cardiology suites along with the randomized clinical trials in 1994 that demonstrated their clinical superiority.^56^, ^57^

### 7.2 Reliability outweighs novelty

Early pacemakers, introduced in the late 1950s, demonstrated the feasibility of long‐term cardiac pacing; however, frequent device failures, often due to battery depletion or lead malfunctions, eroded clinical trust. Over the following decades, incremental progress and iterative refinements in power supply, hermetic sealing, and lead design transformed these fragile prototypes into robust, reproducible devices. Only when reliability was achieved did widespread clinical adoption follow. For milli/microrobots, establishing feasibility and demonstrating clinical value will likely follow a similar arc of incremental improvements that gradually establish clinical trust.

### 7.3 Clear patient benefit ultimately drives acceptance

Adoption of cochlear implants illustrates how even transformative technologies can face deep skepticism before adoption. When first introduced in the late 1970s and 1980s, many clinicians argued that the few electrodes in early devices could never reproduce intelligible speech, regulators worried about safety, and the Deaf community resisted what was perceived as an attempt to “cure” deafness.^58^ For more than a decade, implants were dismissed as experimental. Only when long‐term studies demonstrated that multi‐channel devices restored meaningful auditory function, enabling speech perception and language development, did perceptions shift and cochlear implants become established as the standard of care.

### 7.4 Adoption requires an ecosystem

The Da Vinci surgical system shows that adoption requires more than technical capability; it needs an ecosystem. When first introduced in the late 1990s, the platform faced widespread skepticism: its cost was prohibitive, many surgeons doubted it offered meaningful advantages over established minimally invasive techniques, and hospitals hesitated to invest in a device with uncertain reimbursement. What ultimately shifted perceptions was not just the robot's dexterity but the company's creation of a supportive ecosystem: standardized surgeon training programs, on‐site technical support, service contracts that guaranteed uptime, and aggressive marketing that framed robotic surgery as cutting‐edge. These non‐technical factors, combined with gradual clinical evidence, created a pathway for integration, transforming Da Vinci from a costly curiosity into a dominant surgical robotics platform.

## 8 CONCLUSION

As milli/microrobots gather critical mass, the field is entering a phase defined by translational development. This phase emphasizes addressing real‐world clinical challenges and advancing the technological readiness of robotic systems for medical use. It marks a shift from the dominant focus on discovery and proof‐of‐concept research toward deliberate, stepwise progress aimed at solving well‐defined unmet medical needs.

From an academic perspective, while successfully advancing through the mTRL stages cannot guarantee clinical or commercial success, it can significantly increase the likelihood of achieving a lasting medical impact. Even when market adoption proves slower or more limited than expected, the translational process yields tangible benefits. It sharpens engineering practice, produces validated preclinical and clinical protocols, and generates regulatory‐ready knowledge that future projects can build further upon. In this way, advancing a milli/microrobot design along a structured technology readiness pathway is not a binary proposition of success or failure, but an investment in a growing technological toolbox for the community. The challenges of translation should not deter researchers; instead, they should view it as a natural extension of fundamental discovery, with the potential to transform small‐scale robotics into meaningful clinical tools aimed at advancing healthcare and improving the quality of life of people on a global scale.

Finally, the insights summarized in this review highlight a broader need within the scientific community: to train researchers who can navigate both academic and clinical worlds. The prevailing technology‐push model in academia, optimizing mechanisms, materials, or algorithms in isolation, rarely translates into clinical impact. Bridging this divide requires exposing junior investigators and faculty alike to the constraints, workflows, and priorities that define hospital practice. By framing failure modes, design dependencies, and translational checkpoints for untethered milli/microrobots, the mTRL pathway offers one high‐level structure for making this connection explicit. Empowering researchers with such translational intuition is essential for moving the field and many adjacent biomedical technologies from concept to patient care.

## AUTHOR CONTRIBUTIONS

**Hakan Ceylan**: Conceptualization, project administration, funding acquisition, writing—original draft, figure preparation, writing—review and editing. **Edoardo Sinibaldi**: Writing—original draft, writing—review and editing. **Sanjay Misra**: Writing—original draft, writing—review and editing. **Pankaj J. Pasricha**: Writing—original draft, writing—review and editing. **Dietmar W. Hutmacher**: Writing—original draft, writing—review and editing.

## CONFLICT OF INTEREST STATEMENT

The authors declare no conflicts of interest.

## Data Availability

All data are available within the main text. Additional data can be provided upon request.
